# The Human Endogenous Circadian System Causes Greatest Platelet Activation during the Biological Morning Independent of Behaviors

**DOI:** 10.1371/journal.pone.0024549

**Published:** 2011-09-08

**Authors:** Frank A. J. L. Scheer, Alan D. Michelson, Andrew L. Frelinger, Heather Evoniuk, Erin E. Kelly, Mary McCarthy, Lauren A. Doamekpor, Marc R. Barnard, Steven A. Shea

**Affiliations:** 1 Medical Chronobiology Program, Division of Sleep Medicine, Brigham and Women's Hospital, Boston, Massachusetts, United States of America; 2 Division of Sleep Medicine, Harvard Medical School, Boston, Massachusetts, United States of America; 3 Center for Platelet Research Studies, Division of Hematology/Oncology, Children's Hospital Boston, Dana-Farber Cancer Institute, Harvard Medical School, Boston, Massachusetts, United States of America; Vanderbilt University, United States of America

## Abstract

**Background:**

Platelets are involved in the thromboses that are central to myocardial infarctions and ischemic strokes. Such adverse cardiovascular events have day/night patterns with peaks in the morning (∼9AM), potentially related to endogenous circadian clock control of platelet activation. The objective was to test if the human endogenous circadian system influences (1) platelet function and (2) platelet response to standardized behavioral stressors. We also aimed to compare the magnitude of any effects on platelet function caused by the circadian system with that caused by varied standardized behavioral stressors, including mental arithmetic, passive postural tilt and mild cycling exercise.

**Methodology/Principal Findings:**

We studied 12 healthy adults (6 female) who lived in individual laboratory suites in dim light for 240 h, with all behaviors scheduled on a 20-h recurring cycle to permit assessment of endogenous circadian function independent from environmental and behavioral effects including the sleep/wake cycle. Circadian phase was assessed from core body temperature. There were highly significant endogenous circadian rhythms in platelet surface activated glycoprotein (GP) IIb-IIIa, GPIb and P-selectin (6–17% peak-trough amplitudes; p≤0.01). These circadian peaks occurred at a circadian phase corresponding to 8–9AM. Platelet count, ATP release, aggregability, and plasma epinephrine also had significant circadian rhythms but with later peaks (corresponding to 3–8PM). The circadian effects on the platelet activation markers were always larger than that of any of the three behavioral stressors.

**Conclusions/Significance:**

These data demonstrate robust effects of the endogenous circadian system on platelet activation in humans—independent of the sleep/wake cycle, other behavioral influences and the environment. The ∼9AM timing of the circadian peaks of the three platelet surface markers, including platelet surface activated GPIIb-IIIa, the final common pathway of platelet aggregation, suggests that endogenous circadian influences on platelet function could contribute to the morning peak in adverse cardiovascular events as seen in many epidemiological studies.

## Introduction

**Figure 1:**
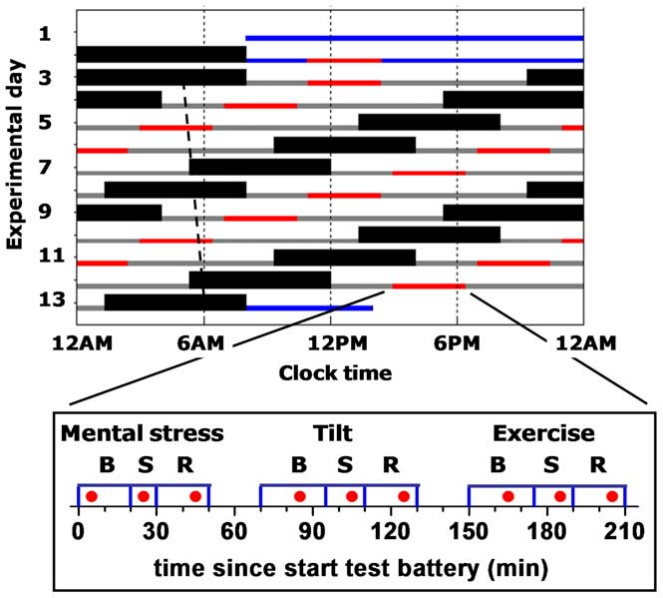
Protocol. Rasterplot, including two baseline days, twelve 20-h cycles, and discharge day of an example subject with a habitual bedtime of midnight. Blue bars, baseline and discharge wake episodes in normal room light (∼90 lux); solid back bars, scheduled sleep (0 lux); gray bars, wake episodes in dim light (∼1.8 lux); red bars, timing of the test batteries; dotted line illustrates the circadian core body temperature minimum throughout the protocol, with an average circadian period of 24.09h in these subjects. Each test battery consisted of a mental, tilt, and exercise stress (S) test, each preceded and followed by a baseline (B) and recovery (R) episode. The timing of the blood draws is indicated as red filled circles.

The risks for major adverse cardiovascular events, including myocardial infarction, stroke, and sudden cardiac death, have daily patterns with peaks in the morning (∼6AM-noon) [1], [2], [3]. These peaks may be related to the morning peak in platelet function [4], which plays a critical role in the formation of an occlusive thrombus [5]. Behavioral stressors such as postural changes upon awakening are known to activate platelets [4], [6]. However, it is also possible that the endogenous circadian timing system—which generates circadian rhythms (∼24-h) in many physiological variables [7] —contributes to this day/night pattern. In the current study, by using a forced desynchrony protocol (**Figure 1**), we tested the hypotheses that: (1) there is an endogenous circadian rhythm in platelet function in humans and (2) there is an endogenous circadian rhythm in the sensitivity of platelet function changes in response to standardized behavioral stressors (mental stress, postural tilt and exercise). We also aimed to compare the magnitude of any effects on platelet function caused by the circadian system with the magnitude of any effects caused by varied standardized behaviors, including mental stress, passive postural tilt and mild exercise. We used both flow cytometry and whole blood aggregability (WBA) testing to assess platelet function.

## Methods

Other physiological aspects of this study have been previously published [7], [8], [9]. The protocol in the current study was an arduous forced desynchrony protocol, comprised of subjects living in the laboratory for 13 days in dim light. Given the intensive nature of the study, we took the opportunity to prospectively test a number of independent hypotheses by making comprehensive measurements across the circadian cycle of platelet function (current manuscript), exercise reactivity [7], passive head up tilt table testing [8], and blood pressure based on combined data from this protocol and two additional protocols [9].

### Subjects

Twelve (6 male; 6 female) healthy volunteers (mean±SD [range]; 25.8±5.7y [20–42y]; BMI: 23.6±3.2 kg/m^2^ [19.9–29.6 kg/m^2^]) completed a 13-day ‘inpatient’ protocol in the Clinical and Translational Research Center at Brigham and Women's Hospital. All subjects gave written informed consent, the studies were approved by Partners Human Research Committee and the studies conformed to the principles expressed in the Declaration of Helsinki. Prior to enrollment, participants underwent extensive screening including medical history, physical and psychological examination, psychological questionnaires, electrocardiogram, and biochemical analysis of blood and urine to confirm that subjects were healthy. To ensure a stable rhythmicity of the circadian system at the time of admission to the laboratory, subjects maintained a regular sleep/wake cycle of 8 h sleep per night for 2–3 weeks immediately prior to admission as verified by sleep/wake diaries, call-in times to a time-stamped voice recorder and wrist actigraphy (Actiwatch; Minimitter, Bend, OR). Subjects reported no shift work for three years or crossing more than one time zone for three months prior to study. To minimize training and detraining effects while in the study, subjects maintained an exercise level equivalent to a 30-min brisk walk per day for the 2–3 weeks immediately prior to admission. Toxicology screens upon admission confirmed that subjects were free of any drugs, including caffeine, alcohol and nicotine.

### Protocol

Subjects spent 13 days and nights in a private laboratory room in constant dim light to avoid resetting the phase of the circadian system (0 lux during scheduled sleep and ∼1.8 lux during scheduled wakefulness), at constant temperature (∼75°F) and free of time cues (**Figure 1**). The environment and many behaviors including physical and mental stresses, sleep, waking and postural changes, can all affect platelet function and were precisely controlled or scheduled. Following two baseline days and nights (16 h of scheduled wakefulness and 8 h of scheduled sleep), subjects completed a forced desynchrony protocol designed to desynchronize the behavioral and circadian cycles, consisting of twelve 20-h ‘days’ (13 h and 20 min of scheduled wakefulness, 6 h 40 min of scheduled sleep; maintaining a 1∶2 sleep:wake ratio) in dim light. Desynchrony between the behavioral and circadian cycles occurs because the 20-h rest/activity cycle in dim light is outside the range of entrainment of the human circadian system, so the circadian system runs free at its inherent rate of close to 24 h [10]. Thus, the sleep and wake episodes as well as all scheduled behaviors were uniformly distributed across the circadian cycle twice (i.e., 2 “beat cycles”). Behavioral test batteries were performed each day, lasted ∼210 min and included three standardized stressors performed in the following order: (i) a mental stressor (10-min serial addition test); (ii) a postural stressor (passive 15-min 60° head-up tilt on tilt table [Model T7605, Metron Medical Australia Pty Ltd, Carrum Downs, Victoria, Australia]); and (iii) an exercise stressor (15-min cycling on an ergometer at 60% maximum heart rate with fixed resistance load cycling at 70 rpm [Cybex Semirecumbent Cycle Ergometer, Division of Lumex, Ronkonkoma, NY]). Each stressor was preceded by a separate 20–25 min baseline period, and followed by a 20-min recovery period. Before each test battery, subjects had been supine for 6 h 40 min during the sleep periods and subsequently, upon lights on, remained in a semi-recumbent posture (upper body at 45°) for 2 h 55 min. Subjects were provided with a urinal or bedpan as needed during that time. During each test session, subjects maintained the same body posture: semi-recumbent during mental stress session, supine during the tilt session excepting the scheduled head up tilt, and seated on the cycle ergometer; were asked to limit any movement excepting the scheduled cycling; and not to talk except in response to structured questions. In between the three test sessions within each test battery there were two 20-min breaks during which subjects were gently slid from bed to tilt table and calmly walked ∼2 m from the tilt table to the cycle ergometer, respectively.

### Circadian Phase Assessment

Each measurement was assigned a circadian phase (0°–359°) based on non-orthogonal spectral analyses of continuous core body temperature recorded throughout the forced desynchrony protocol, with the fitted core body temperature minimum set to 0°, as previously published [10]. To facilitate translation of circadian phase to clock time, we report “corresponding clock time”, defined as the group-average clock time at which a circadian phase would have occurred under normal entrained conditions i.e., with subjects sleeping at their habitual times during the night, and awake during the day (in these subjects, 0° occurred at ∼4:30 AM; 60° occurred at ∼8:30 AM, etc.).

### Blood Sampling

Blood was sampled at 20-minute intervals across the test batteries via a 12-foot intravenous catheter with heparin infusion rate of ∼200 IU/h: during each baseline, stress and recovery section of each of the three stress tests (mental stress test, tilt table test and exercise test; see **Figure 1** for details on timing of blood samples). For WBA, blood samples were assayed from each of the 12 test batteries (each of the twelve 20-h ‘days’) for all subjects. For flow cytometry assays (including platelet count) blood samples were assayed from the 6 test batteries during the second half of the forced desynchrony protocol, thus spanning a full circadian cycle, for eleven subjects (technical difficulties precluded analysis of data in the other subject). As opposed to 9 blood samples per test battery being used for all other variables, for ATP release, only the first blood sample during each of the 12 test batteries (mental baseline) was assayed for ten subjects (technical difficulties precluded analysis of data in the other two subjects).

### Flow Cytometry

Blood samples were assayed by whole blood flow cytometric detection of platelet surface GPIb alpha, P-selectin, and activated GPIIb-IIIa (reported by the binding of monoclonal antibody PAC1) [11]. Monoclonal antibody PAC-1 recognizes only the conformationally activated form of GPIIb-IIIa [12]. The specific commercially available monoclonal antibodies used were fluorescein isothiocyanate (FITC)-conjugated PAC-1 (Becton Dickinson), phycoerythrin (PE)-conjugated anti-P-selectin (DAKO-Cytomation), and PE-Cy5 conjugated anti-GPIb (BD-Pharmingen). Samples were analyzed in a FACSCalibur (Becton Dickinson) flow cytometer calibrated daily with Spherotech RCP-30-5 calibration beads to assure consistent fluorescence measurements. Platelet surface activated GPIIb-IIIa was expressed as percent positive platelets; platelet surface P-selectin and GPIb were expressed as mean fluorescent intensity (MFI). Forward light scatter in the flow cytometer was used as an approximate estimate of platelet size (with increased forward light scatter signifying increased size of platelets) [13].

### Whole blood platelet aggregability (WBA)

Whole blood samples (1 mL) were collected in sodium citrate tubes. Each blood sample was diluted 1∶1 [450 µL whole blood and 450 µL of physiological saline (NaCl)] at 37°C. Platelet aggregability was assessed in duplicate by stimulation with 5 µg/mL collagen by dual channel whole blood/optical lumi aggregometer (560VS, Chrono-Log Corporation, Havertown, PA) and analyzed with Chrono-Log software (Aggro/Link for Windows). Platelet aggregability was quantified by the area under the curve of the impedance signal across 10 min immediately following stimulation with collagen and reported in Ohm*sec. For quality control, data were excluded if duplicates differed by more than 12.5% from the mean (excluding 18% of data), resulting in a CV of 7%. ATP release from platelets was assessed by the addition of 100 µl of Chrono-lume solution (0.16 mg/ml luciferin and 17,600 U/ml d-luciferase). All WBA measurements were completed within 20 min of the blood draw.

### Statistical Analysis

Based on pre-hoc criteria to eliminate likely ‘spurious outliers’, values more than 3 standard deviations from the mean within each subject across all wake periods were excluded from the analysis. Thereafter, the effects of the circadian cycle and the behavioral stressors of the test batteries were assessed by cosinor analyses using mixed model ANOVA's for each variable including a fundamental circadian component (∼24-h), a harmonic component (∼12-h), and the 9 test conditions (baseline, exercise and recovery for mental stress, postural stress, and exercise stress test). Tukey's range tests were performed to assess changes across the test battery. All statistical analysis was performed by JMP (SAS Institute) and significance was set at p<0.05.

## Results

The average circadian period estimated from core body temperature measurements was 24.09±0.22h [mean±SEM; range: 23.8–24.6h]. There were no significant interactions between circadian phase and time into the behavioral test battery (mental stress, postural tilt and exercise); so all reported statistics are based on mixed model ANOVA's across circadian phase without interaction.

### Effect of Circadian Cycle (independent of changes in behavior and environment)

**Figure 2:**
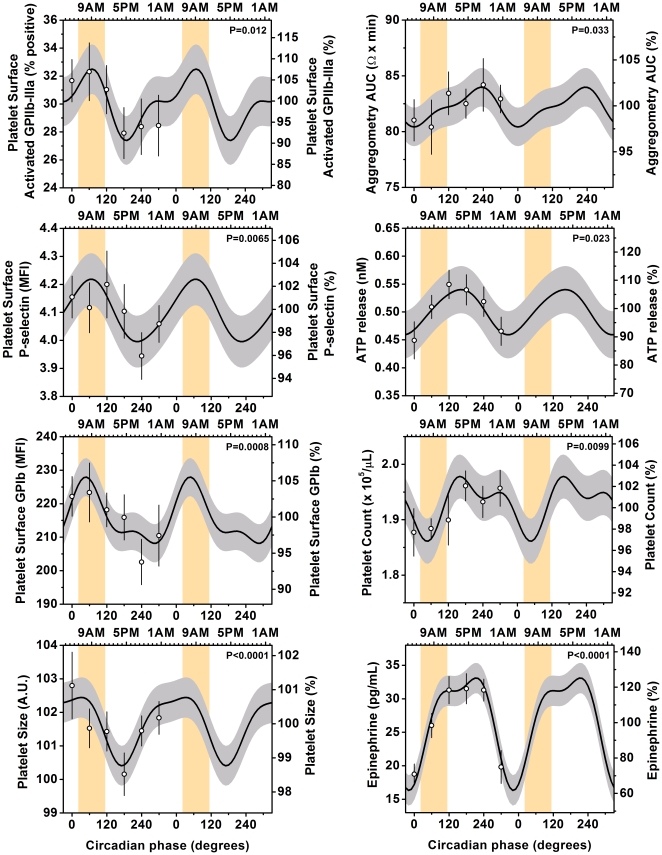
Independent influence of circadian cycle on platelet function. Cosinor models of circadian rhythms in platelet function. Cosinor and other statistical analysis were performed on the 360° data sets, whereas data are double plotted (2 identical circadian cycles) to aid visualization of rhythmicity (the second circadian cycle data were not used for analyses). Each of the platelet surface markers of platelet activation (activated GPIIb-IIIa, P-selectin, and GPIb) and platelet size had significant endogenous circadian rhythms with circadian peaks corresponding to the vulnerable time of 6AM-noon (indicated by the orange bars). Platelet count, ATP release, WBA and plasma epinephrine also had significant circadian rhythms but with peaks later in the circadian cycle (3–8PM). The cosine models (black lines) and 95% confidence intervals (gray areas) are based on mixed model analyses and use precise circadian phase data. To show that these models adequately fit the actual data, we also plot average data grouped into 60 circadian degree windows with SEM error bars (averaged data are not double plotted). Bottom x-axes, circadian phase with 0° indicating the timing of the core body temperature minimum (average ∼4:30AM in these subjects); top x-axes, corresponding average clock time in these subjects; left y-axes, absolute values; right y-axes, percentage of each individual's mean across the protocol; P values, significance of circadian effect from cosinor analyses.

For the group, there were highly significant endogenous circadian rhythms in platelet surface activated GPIIb-IIIa, platelet surface GPIb, platelet surface P-selectin, and platelet size (p always ≤0.01), with circadian peaks corresponding to the epidemiologically identified vulnerable period of ∼6:30-9AM, (**Figure 2** and **Table 1** **)**. The peak-to-trough circadian amplitudes ranged from 6-17% for the platelet surface antigens and 2% for estimated platelet size. Platelet count, ATP release, platelet whole blood aggregability and plasma epinephrine also had significant circadian rhythms but with later peaks (3-8PM). Although the circadian peak in plasma epinephrine occurred after the vulnerable morning period, the fastest rate of increase occurred during the vulnerable period (**Figure 2**).

**Table 1:** Endogenous circadian rhythms in platelet function from cosinor analyses.

Variable	P-value circadian	P-value harmonic	Timing peak (degrees)	Timing peak (clocktime)	Peak-to-trough amplitude	Peak-to-trough amplitude (% of mean)
GPIIb-IIIa	0.012	n.s.	70°	∼9AM	5.1% pos	17%
P-selectin	0.0065	n.s.	70°	∼9AM	0.22 MFI	6%
GPIb	0.0008	n.s.	50°	∼8AM	19.8 MFI	9%
Estimated platelet size	<0.0001	n.s.	30°	∼6:30AM	2.0 A.U.	2%
Aggregability	0.033	n.s.	230°	∼8PM	3.6Ω*min	4%
ATP release	0.023	n.s.	160°	∼3PM	0.08nM	16%
Platelet count	0.0099	n.s.	160°	∼3PM	0.12×10^5^/µL	6%
Epinephrine	<0.0001	0.0087	210°	∼6:30PM	17 pg/mL	68%

### Effect of Mental Stress, Postural Tilt and Exercise (independent of circadian phase)

**Figure 3:**
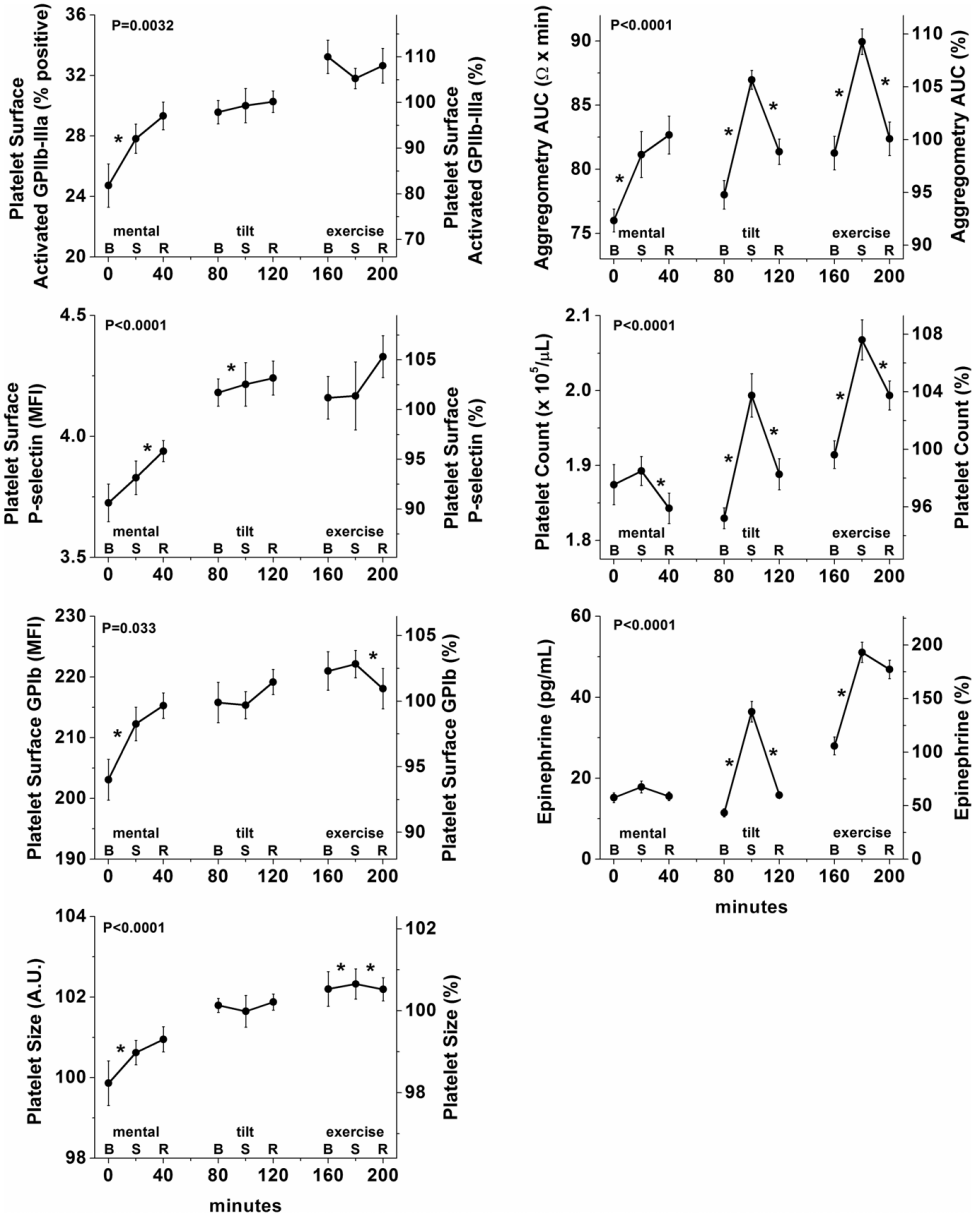
Independent influence of behavioral stressors on platelet function. WBA, platelet count, and plasma epinephrine were increased by each of the three stressors, with a (partial) recovery during recovery. In contrast, while the platelet surface markers showed a gradual increase across the approximately 3-h test battery, there was no consistent increase and recovery caused by the three stressors. Mental, mental stress test; tilt, passive head up tilt table test; exercise, cycle exercise test; B, baseline; S, stress test; R, recovery; x-axes, minutes from first blood sample within test battery; left y-axes, absolute values; right y-axes, data expressed as a percentage of each individual’s mean values across the forced desynchrony protocol; error bars, SEM; P-values, significance for effect of time across full stress test battery (9 time points); *, significance for change between consecutive samples (from baseline to stress test and from stress test to recovery). Note platelet ATP release is not shown (see above text).

WBA, platelet count, and plasma epinephrine significantly varied across the standardized test battery (all P<0.0001; **Figure 3, right panel** **s**), due to an increase during exposure to the three behavioral stressors usually with incomplete recovery between subsequent stressors. Mental stress generally had the smallest effects (6% for WBA, n.s. for platelet count and plasma epinephrine), while the effects of tilt and of exercise were larger (averaging 11% for WBA, 9% for platelet count, and 91% plasma epinephrine). We could not test for an effect of the test battery on platelet ATP release because this was assessed only during resting baseline conditions. Platelet surface activated GPIIb-IIIa, P-selectin, GPIb, and platelet size increased gradually across the test battery (all P<0.05; **Figure 3, left panel** **s**), increasing from mental baseline to mental recovery, for all four flow cytometry measures (all P<0.05), but without consistent changes across the tilt table test or the exercise test.

### Comparison of Effects of Circadian Cycle and Behavioral Stressors

The magnitudes of the circadian effects (peak-to-trough difference; **Table 1**) on the platelet activation markers were always larger than the effects of any of the three separate stressors: GPIIb-IIIa (circadian: 17% vs. behavioral stressors: max 10%), P-selectin (6% vs. max 3%), GPIb (9% vs. max 4%), and estimated platelet size (2% vs. max 1%). The magnitudes of the effects of the circadian system on platelet count (6%) and plasma epinephrine (68%) were comparable to the magnitudes of the effects of the three stressors (platelet count: 1–9%; epinephrine: 10–94%). Only for platelet aggregability was the circadian influence smaller than that of the stressors (4% vs. 6–11%).

## Discussion

Our results reveal a potentially important influence of the circadian system on platelet function in humans. Many previous studies have investigated the day/night patterns in platelet function [4], but ours is the first to determined the influence on platelet function of the circadian system separate from the influences of the changes in behavior and environment that normally occur across the day and night, including changes in sleep/wake state, supine/upright posture, rest/activity, fasting/feeding, and the light/dark cycle [7].

### Potential clinical relevance

A better understanding of the relative importance of circadian rhythms and behaviors on platelet function is important especially for the increasing number of shift workers, travelers across time zones, and people with sleep disorders in whom influences of the circadian system are uncoupled from those of their behaviors. Shift workers, including even permanent night workers, typically experience chronic and/or recurrent misalignment between the circadian system and the sleep/wake cycle [14], [15]. Also sleep disorders, especially circadian rhythm sleep disorders, are associated with chronic and/or recurrent circadian misalignment [16]. In jet lag, the misalignment is transient, although the number of days required for reentrainment may depend on the organ systems involved [17]. The absence of statistical interaction between circadian phase and the behavioral stressors suggests that the effect of the circadian system and behavioral stressors on platelet function are additive. The magnitude of the circadian effect was always larger than that of any of the three separate stressors for all platelet surface markers of platelet activation. Given that shift workers are at increased risk for cardiovascular events [18] this larger effect of the circadian system as compared to behavioral stressors on platelet activation may warrant future studies to determine whether antiplatelet therapy in shift workers can be further optimized by basing the timing of therapy on their internal circadian phase rather than on their behavioral sleep/wake cycle. The three measured surface markers of platelet activation peaked at an endogenous circadian phase corresponding to 8–9AM, consistent with our hypothesis of the presence of an endogenous circadian rhythm in platelet function peaking around the vulnerable time for major adverse cardiovascular events (6AM-noon [1], [2], [3]). This is of likely clinical importance as these factors represent measures of the final common pathway of platelet aggregation (GPIIb-IIIa) [12]; adhesion of activated platelets to monocytes and neutrophils (P-selectin) [19]; and adhesion of activated platelets to subendothelial collagen in the blood vessel wall (GPIb) [20]. Indeed, FDA-approved drugs (abciximab, integrilin and tirofiban) that block GPIIb-IIIa are of benefit as antithrombotic therapy in acute coronary syndromes [21], and in animal models, antagonism of platelet surface P-selectin [22] and platelet surface GPIb [23] has antithrombotic benefit. The later circadian peaks in aggregability (representing platelet reactivity), platelet count and ATP release suggest that these may not be as relevant to the day/night pattern of increased risk for thromboembolistic events in the morning as the measures of platelet size and platelet surface activated antigens (GPIIb-IIIa, P-selectin, surface GPIb), which represent *in vivo* circulating activated platelets.

### Potential mechanisms involved in circadian variation in platelet function

The circadian rhythms in circulating platelet function could be caused by circadian rhythms in three general mechanisms: (i) humoral factors, including hormones (e.g., epinephrine, norepinephrine, melatonin and cortisol), endothelial-derived factors (e.g., nitric oxide, prostacyclin), platelet-derived factors (e.g., ATP), and other blood-borne substances (e.g., fibrinogen, factor VII, 5-hydroxytrypamine, vasopressin); (ii) release or sequestering of platelets by the spleen, liver, lungs, and/or bone marrow, which is controlled by humoral factors (e.g., epinephrine [24]); and/or (iii) intrinsic circadian oscillations within the anucleate platelets [25]. Indeed, although mechanisms for intrinsic circadian oscillations in platelets are unknown, circadian rhythms that are independent of transcription-translation feedback loops have recently been demonstrated in human red blood cells that are also anucleate and involve redox cycles of peroxiredoxins [26]. Humoral factors that express large-amplitude endogenous circadian rhythms and that could drive the endogenous circadian rhythm in platelet function include cortisol, epinephrine, norepinephrine, and melatonin that have a concurrent peak (cortisol [7], [27]), rapid rise (epinephrine [**Figure 2**] and norepinephrine [7]), or rapid fall (melatonin [28], [29]) at the time of the circadian peak in the platelet surface markers of platelet activation. Future studies are required to investigate the relative influence of these circadian hormones on platelet function and to determine whether or not other humoral factors show relevant endogenous circadian rhythms and phases, independent of the behavioral sleep/wake and fasting/feeding cycle.

Differences in timing of the endogenous circadian peaks between the platelet surface markers and WBA may be explained in part by the different circadian timing between platelet count and platelet surface markers, because platelet surface markers (as measured here on individual platelets) are not influenced by changes in platelet count, while WBA is increased with increasing platelet count. Alternatively, there may be a ‘ceiling effect’ in the aggregometry assay since platelets that are already partially activated *in vivo* at 9AM (as assessed by platelet surface activation markers) may be refractory to further *ex vivo* activation by collagen during WBA [30].

The endogenous circadian rhythms in platelet activity and platelet aggregability are consistent with recent data showing a direct effect of core clock genes, such as *Bmal1*, *Clock* and *Per2*, on the activity of key factors in hemostasis, such as von Willebrand factor and megakaryocytes [31], [32], [33]. Furthermore, these recent data demonstrate that disruption of the positive limb of the primary transcription-translation feedback loop driving circadian oscillations (BMAL1/CLOCK) leads to a prothrombotic phenotype while disruption of the negative limb (e.g., *Per2*) leads to a hemorrhagic phenotype.

### Potential mechanisms involved in effects of behavioral stressors on platelet function

Tilt and exercise, but not mental stress, caused an increase in platelet count simultaneously with large increases in epinephrine (**Figure 3**). The increase in catecholamines may explain—at least in part—the observed changes in platelet count, because catecholamines can stimulate immediate release of platelets from the spleen [24]. The increase in platelet count may subsequently help explain the simultaneous increase in platelet aggregability in response to these behavioral stressors. On the other hand, the platelet surface makers did not consistently increase specifically during the tilt and exercise tests, nor decrease during the recovery phases following these stressors. The absence of a clear effect of postural stress (i.e., passive head-up tilt) on platelet surface markers of platelet activation and the presence of a clear increase in WBA, platelet count, and epinephrine in response to the head-up tilt is consistent with a previous study that examined the effect on platelets of the transition from lying to standing [34]. Also the absence of a clear effect of exercise on surface markers of platelet activation is consistent with a previous study by our group in which exercise failed to cause consistent changes in platelet activation in physically active subjects [6]. Interestingly, in that same study, we showed that exercise by sedentary subjects did result in significant platelet activation [6].

### Therapeutic considerations

There is clear evidence that the presently measured markers of platelet activation are clinically relevant. FDA-approved drugs (abciximab, eptifibatide and tirofiban) that block GPIIb-IIIa (the platelet receptor that we specifically measured by flow cytometry and the receptor upon which whole blood aggregometry is specifically dependent [35]) are of benefit as antithrombotic therapy in acute coronary syndromes [21]. In animal models, antagonism of platelet surface P-selectin [22] and platelet surface GPIb [23] has antithrombotic benefit. A better understanding of the relative importance of circadian rhythms and behaviors on platelet function may help reveal new therapeutic targets for cardiac patients. Ultimately, chronotherapy could be designed to specifically target pharmacological or behavioral interventions to those time windows of greatest risk for cardiovascular events. Rather than the current clinical practice of attempting to maintain therapeutic levels of antiplatelet drugs throughout each day and night or base medication timing on presumed patient convenience, it may be therapeutically beneficial for antiplatelet agents to specifically target the circadian phases of greatest platelet aggregability to reduce thrombotic complications, while minimizing hemorrhagic complications during periods of reduced platelet aggregation [36].

### Limitations and Strengths

Limitations of this initial study of the endogenous circadian rhythmicity in platelet function include: inclusion of healthy controls rather than individuals at risk for cardiovascular events; small group size; and no assessment of fibrinolytic activity. We also note that the blood sampling technique incorporating a 12-foot i.v. line with heparin infusion is non-standard in flow cytometry studies, and conceivably could have affected some platelet function measures—although any such effects are likely to be consistent across the protocol and would not explain the observed changes across the circadian cycle. Circadian analysis of a subset of the WBA and platelet count data (i.e., data at rest immediately before exercise and data during exercise) has previously been published [7] and showed a ∼12-h (circadian harmonic) rhythmicity for WBA and no rhythmicity for platelet count. The increase in statistical power by inclusion of ∼4.5 times more data in the current analysis may help explain why we now find significant circadian rhythmicity for both WBA (P = 0.03) and platelet count (P = 0.01). In addition, we cannot exclude the possibility that the effect of the circadian system and behavioral state interact, although such putative interaction did not reach statistical significance in the current study. The strengths of the study include: the forced desynchrony protocol allowing the systematic assessment of effects of the human endogenous circadian system on platelet function—independent of changes in environment and behavior; the highly controlled laboratory environment; an intensive within-subject design with frequently repeated measures over 13 consecutive days; and the comprehensive assessment of platelet function, including various estimates of the *in vivo* state of platelet activation (activated platelet surface GPIIb-IIIa, platelet surface P-selectin, platelet surface GPIb), *in vitro* platelet reactivity (WBA and ATP release), and platelet count. These results represent a valuable ‘reference’ data set in healthy young people, critical for comparison with vulnerable populations.

### Conclusion

The present data show a robust influence of the endogenous circadian system on platelet function independent of the sleep/wake cycle. If these data collected in healthy individuals can be extrapolated to more vulnerable populations, this would suggest that the endogenous circadian rhythms in platelet surface activated GPIIb-IIIa, platelet surface P-selectin, and platelet surface GPIb may play a causal role in the morning peak in adverse cardiovascular events. Thus, future circadian studies are required to investigate the responsible circadian signals underlying the circadian rhythms in platelet activity and whether there are differences in the timing and magnitude of these circadian effects in vulnerable populations, such as subjects who are older, sedentary, or obese and patients with coronary artery disease, prior myocardial infarction, or heart failure.
